# Association between emergency department length of stay and adverse perioperative outcomes in emergency surgery: a cohort study in two Colombian University hospitals

**DOI:** 10.1186/s12873-019-0241-6

**Published:** 2019-04-17

**Authors:** Félix R. Montes, Skarlet Marcell Vásquez, Claudia Marcela Camargo-Rojas, Myriam V. Rueda, Lina Góez-Mogollón, Paula A. Alvarado, Danny J. Novoa, Juan Carlos Villar

**Affiliations:** 1 Departamento de Anestesiología, Fundación CardioInfantil– Instituto de Cardiología and Universidad del Rosario, Bogotá, Colombia; 2 Grupo de Cardiología Preventiva, Facultad de Ciencias de la Salud, Universidad Autónoma de Bucaramanga, Bucaramanga, Colombia; 3 Departamento de Investigaciones, Fundación CardioInfantil– Instituto de Cardiología, Bogotá, Colombia; 4 Departamento de Medicina Interna, Facultad de Ciencias de la Salud, Universidad Autónoma de Bucaramanga and Fundación Oftalmológica de Santander- Clínica FOSCAL, Bucaramanga, Colombia; 5 Departamento de Medicina Interna, Fundación CardioInfantil– Instituto de Cardiología and Universidad del Rosario, Bogotá, Colombia

**Keywords:** Emergency surgery, Emergency department, Length of stay, Outcome

## Abstract

**Background:**

In low- and middle-income countries emergency surgery represents a higher proportion of the total number of surgeries and is associated with greater morbidity/mortality. Study aims were to determine if emergency department length of stay (ED-LOS) was associated with adverse perioperative outcomes and if such association varied across patient’s risk categories.

**Methods:**

A retrospective cohort study was conducted of adult patients who underwent orthopedic or abdominal emergency surgery at two Colombian University hospitals. The population comprised a mix of a representative sample of eligible cases, with unselected patients (2/3), enriched with a high-risk subset (1/3). ED-LOS was defined as the interval between emergency department arrival and surgery start time. Our primary outcome was an adverse perioperative outcome during hospitalization, which was a composite of in-hospital mortality or severe complications such as major cardiovascular adverse events, infection, renal failure and bleeding.

**Results:**

Among 1487 patients analyzed, there were 519 adverse perioperative outcomes including 150 deaths. In the unselected sample (*n* = 998) 17.9% of patients presented an adverse perioperative outcome with a mortality of 4.9%. The median ED-LOS was 24.6 (IQR 12.5–53.2) hours. ED-LOS was associated with age, comorbidities and known risk factors for 30-day mortality. Patients developing an adverse perioperative outcome started surgery 27.1 h later than their counterparts. Prolonged ED-LOS increased the risk of an adverse perioperative outcome in patients without risk factors (covariate-adjusted OR = 2.52), while having 1–2 or 3+ risk factors was negatively associated (OR = 0.87 and 0.72, respectively, *p* < 0.001 for the interaction).

**Conclusion:**

Prolonged ED-LOS is associated with increased adverse perioperative outcome for patients without risk factors for mortality, but seems protective and medically justified for more complex cases.

## Background

Emergency surgery (ES) is associated with significantly higher morbidity and mortality when compared with elective procedures [1–4]. Factors that influence outcomes after ES include patient’s previous clinical condition [5, 6], the availability of health care resources [7], and the timeliness of administrative and organizational processes [8].

In developing countries, ES represents a relatively high fraction of the total surgical procedures [9]. International comparisons indicate that in countries with lower scores in the United Nations human development index, mortality from ES is 2 to 3 times higher, compared to the counterparts [10]. In recent years, as a result of the distribution and access to health services, hospitals in Colombia have experienced increasing demands and overcrowding in emergency departments (ED) [11]. A large international study reported that ES represented 11% of all non-cardiac surgeries, which contrasts with data from Colombian centers that participated in such study, where a significantly higher ES rate (43%) was found and associated with a 3.5-fold increase in 30-day mortality [3].

Although many predictors of postoperative morbidity and mortality are not modifiable, there still certain opportunities for improvement in the process of patient care that could alter these results. The ED length of stay (LOS), a potentially modifiable factor, is linked with worse patient outcomes in specific populations. A prolonged ED-LOS has been associated with an increase in 30-day mortality in critically ill patients [12, 13], and patients with traumatic injuries [14]. These patients require highly specialized care, often protocolized and administered on a one-by-one basis, which is difficult to provide for the frequently busy ED staff [12, 14]. However, there is little understanding of the relationship between ED LOS and outcomes in patients admitted for ES, particularly in developing countries.

Study aims were to determine if ED LOS was associated with the incidence of adverse perioperative outcomes (APO) and whether this association varied across the patient’s risk categories in patients undergoing ES.

## Methods

### Study design and setting

We conducted a retrospective cohort study, in accordance with the “Strengthening the Reporting of Observational Studies in Epidemiology” (STROBE) statement [15]. Study population comprised representative samples of non-cardiac surgery patients at two university hospitals in Colombia. Eligible patients were those admitted to the ED of both participant centers, aged 45 or more, who subsequently underwent non-elective (i.e., urgent/emergent) orthopedic or abdominal surgical procedures. The time window for identification and data collection went from January 1, 2012 to June 30, 2017.

Because of the resources and staff available for this project, center 1 was to provide roughly twice the participants included at center 2. While keeping representativeness, we also sought to generate more precise estimates of the association of interest by ensuring the inclusion of sufficient patients across all risk levels and a pre-defined number of adverse perioperative events. This goal was sought by enriching representative samples with subsets of higher risk populations within each hospital.

Our population thus included two patient subgroups (2:1 ratio) at both hospitals within the time window for patient screening: Two thirds of our population came from representative samples (all eligible patients admitted at randomly-selected weeks at center 1 or consecutively within a time period for center 2). The remaining third were patients whose hospital stay or costs exceeded the 75th percentile of the representative samples at each hospital. This second subset was selected in similar fashion, until recruitment goals were reached (See statistical methods/ sampling size below).

Eligible procedures included any unplanned operation linked to admissions via EDs, requiring general or regional (epidural or spinal) anesthesia and one or more nights of hospital stay. We excluded semi-elective procedures (those in patients initially admitted to the ED, then discharged without a procedure, but readmitted later for elective surgery), reoperations and trauma cases.

The study centers were two large (over 200-bed) University Hospitals in Colombia (Fundación Cardioinfantil – Instituto de Cardiología in Bogotá, and Fundación Oftalmológica de Santander - Clínica FOSCAL in Bucaramanga). These hospitals provide acute surgery services 24/7 in a wide number of specialties (over 1000 non-cardiac, non-elective surgeries annually). The institutional review boards (research and ethics) of both centers approved the study protocol (Approval certificates code 441 June 11st 2014 for Fundación Cardioinfantil – Instituto de Cardiología, and code 53 July 28th, 2016 for Fundación Oftalmológica de Santander - Clínica FOSCAL) and because of its nature waived the need for individual informed consent.

### Study variables and data collection

The exposure of interest was ED-LOS, the interval between ED arrival and surgery start time. The primary endpoint was the incidence of APO during hospitalization, which was a composite of in-hospital mortality, major cardiovascular adverse events, infection, renal failure, and severe bleeding. Major cardiovascular adverse events included myocardial infarction, cardiac arrest, stroke, deep vein thrombosis, and pulmonary embolism. In-hospital mortality alone was used as a secondary endpoint.

Electronic medical records were the primary data source at both hospitals. Trained research assistants manually extracted patient characteristics, past medical history, including known perioperative predictors of 30-day mortality, as reported in the VISION study [3], and current medications. We also registered medical procedures before surgery, including any medical assessment (from medical/surgical specialties), diagnostic lab or images requested. In addition, we obtained data on the index surgical procedure and emerging perioperative complications until discharge. Finally, costs were obtained by reviewing all hospital invoices/medical bills to the insurance companies (third payer’ perspective).

Data quality control included a number of actions. Firstly, we had limits and cross-validation checks in the data entry forms. Secondly, we examined data correlations throughout by centers and partial splits of the data. For outcome data, we have a second assessor cross-reviewing all non-fatal events. A third, independent assessor re-adjudicated any discrepancy so that all recorded events needed two concordant opinions to remain in the database.

### Statistical aspects

Sampling size: We aimed at detecting a meaningful association (RR ≥ 1.5) for exposed individuals (25% of the population). That is, we hypothesized that the population in the longest quart of ED-LOS was at least 1.5 times more likely to develop severe perioperative complications. Assuming a 15% baseline risk for the primary outcome, including at least 1700 patients would give us over 90% power to detect such an association (alpha level 5%). For the analysis of ortality, assuming a 5% rate (based on the VISION data for Colombia), the same sample allowed 90% power to detect an RR ≥ 2. Regarding events, the goal was to record over 100 in-hospital deaths (and around 300 severe complications), admitting multivariate models up to 7 predictors (10–15 events for predictor) as recommended for death [16].

In order to achieve our sample size goals, we devised a three-step sampling process. Seeking efficiency, we decided to enroll approximately 1500 patients but ensuring the expected number of events by over-representing the high-risk portion (with roughly 1/3) of the study population. First, we selected all eligible, consecutive patients from randomly selected months within the time window (to reach approximately 500 patients among both centers). This representative sample allowed us to identify a) relevant inter-center differences for future sampling/analyses and b) the 75th percentile of hospital stay or costs (as a marker of a high-risk population for sampling). The second step involved a similar sampling within the subset above these cut-off points to enrich the cohort with (about 500 patients from) high-risk population. A third, additional subset involved a parallel sample of consecutive patients recently discharged at the coordinating center, recorded on a monthly basis, up to completion of the sample.

Statistical Analysis: Baseline demographics and clinical characteristics were summarized using descriptive statistics. For inter-group comparisons of continuous variables, we used Student’s t-tests, or the Mann-Whitney U test/ Wilcoxon signed rank according to their distribution. For categorical variables, we used either a Chi-square test or Fisher’s exact test, as determined by cell frequencies. In a two-step process, we first explored variables potentially associated with both adverse perioperative outcomes and mortality. Those with *p* values< 0.20 were considered potential confounders. To control for these variables, we included them as factors in a multivariable logistic regression model to identify independent associations. The models included an interaction term to evaluate a potential effect modification between ED-LOS and perioperative predictors of 30-day mortality for the outcome. We reported adjusted odds ratios along with their 95% confidence intervals and associated *p*-values. All statistical analyses were performed using statistical software Stata/SE 14.2.

## Results

**Fig. 1:**
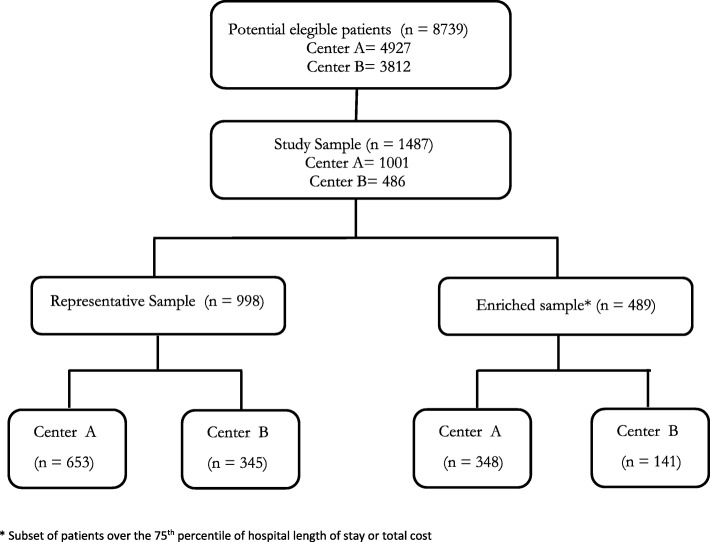
Study flow chart

Overall, our analyses include 1487 patients (see Fig. 1), 2/3 from representative samples, with center A providing 2/3 of the population. Table 1 shows patient demographics and preoperative characteristics using the representative samples of both centers. Although center A admitted more often complex patients (with prior medical conditions or cumulated risk factors, receiving more frequent assessments and diagnostic workup), ED-LOS were similar among centers (median time 24.2 and 24.8 h, *p* = 0.965).

**Table 1:** Patient characteristics of representative sample

	Total (*n* = 998)	Center A (*n* = 653)	Center B (*n* = 345)	*P*
Gender, male	5111 (51.2)	353 (54.1)	158 (45.8)	0.013
Age (years)	62.4 (53.2–73.5)	62.4 (53.2–73.5)	62.3 (53.4–73)	0.797
Preoperative medical conditions				
Hypertension	396 (39.7)	278 (42.69	118 (34.2)	0.010
Diabetes mellitus	137 (13.3)	89 (13.6)	48 (13.9	0.901
Chronic renal disease	59 (5.9)	43 (6.6)	16 (4.6)	0.215
Major cardiovascular disease^b^	252 (16.9)	200 (19.9)	52 (10.7)	< 0.001
COPD	62 (6.2)	51 (7.8)	11 (3.2)	0.014
Active cancer	197 (13.3)	154 (15.4)	43 (8.9)	< 0.001
Major general surgery	236 (15.9)	179 (17.9)	57 (11.7)	0.002
Risk factors for 30-day mortality^a^				0.006
No risk factors	434 (43.5)	275 (42.1)	159 (46.1)	
1 or 2 risk factors	433 (43.4)	276 (42.3)	157 (45.5)	
≥ 3 risk factors	131 (13.1)	102 (15.6)	29 (8.4)	
Preoperative interventions				
Medical assessments	6 (4–10)	7 (5–11)	4 (3–7)	< 0.001
Laboratories	8 (4–16)	11 (6–19)	6 (3–11)	< 0.001
Diagnostic images	2 (1–3)	2 (1–4)	2 (1–3)	< 0.001
Type of surgery				< 0.001
General	735 (73.6)	522 (79.9)	213 (61.7)	
Orthopedic	263 (26.4)	131 (20.1)	132 (38.3)	
ED LOS (hours)	24.6 (12.5–53.2)	24.2 (13.2–50.5)	24.8 (11–64.3)	0.965
Duration of surgery (hours)	1.6 (1.3–2.3)	1.8 (1.3–2.4)	1.5 (1.1–2)	< 0.001

Data are presented as median (interquartile range), or absolute number (%)

*PVD* Peripheral vascular disease, *COPD* Chronic obstructive pulmonary disease, *ED* Emergency department, *LOS* Length of stay

^a^Risk factors as defined by VISION study [3]: Age ≥ 65 years, history of coronary artery disease, congestive heart failure, PVD, stroke, COPD, active cancer and major general surgery

^b^Includes history of coronary artery disease, stroke, heart failure, peripheral vascular disease

Using 48 h as working cut-off, ED-LOS had a positive relationship with age, comorbidities and 30-day mortality risk factors present on admission (Table 2). ED-LOS was also associated with having more frequent medical assessments or diagnostic workup in the ED, performing abdominal surgery and the length of the procedure itself. Of note, 141 (24.2%) of patients who stayed in the ED > 48 h had no 30-day mortality risk factors.

**Table 2:** Patient characteristics according to emergency department length of stay

	ED-LOS ≤ 48 h (*n* = 905)	ED-LOS > 48 h (*n* = 582)	*P*
Gender, male	469 (51.8)	294 (50.5)	0.622
Age (years)	62.6 (53.1–73.6)	68.4 (57.8–77.2)	< 0.001
Medical center			0.481
Center A	603 (60.2)	398 (39.8)	
Center B	302 (62.1)	184 (37.9)	
Preoperative medical conditions			
Hypertension	361 (39.9)	307 (52.8)	< 0.001
Diabetes mellitus	121 (13.4)	137 (23.6)	< 0.001
Chronic renal disease	53 (5.9)	69 (11.9)	< 0.001
Major cardiovascular disease^a^	118 (13.0)	134 (23.0)	< 0.001
COPD	57 (6.3)	62 (10.7)	0.002
Active cancer	76 (8.4)	121 (20.8)	< 0.001
Major general surgery	132 (14.6)	104 (17.9)	0.090
Risk factors for 30-day mortality^b^			0.006
No risk factors	377 (41.7)	141 (24.2)	
1 or 2 risk factors	403 (44.5)	289 (49.6)	
≥ 3 risk factors	125 (13.8)	152 (26.1)	
Preoperative interventions			
Medical assessments	5 (4–7)	16 (10–30)	< 0.001
Laboratories	8 (4–14)	27.5 (12–60)	< 0.001
Diagnostic images	2 (1–3)	4 (2.5–7)	< 0.001
Type of surgery			< 0.001
General	700 (77.3)	403 69.2)	
Orthopedic	205 (22.7)	179 (30.8)	
Duration of surgery (hours)	1.8 (1.3–2.4)	2 (1.3–2.8)	< 0.001

Data are presented as median (interquartile range), or absolute number (%). *ED-LOS* Emergency department length of stay, *COPD* chronic obstructive pulmonary disease

^a^Includes history of coronary artery disease, stroke, heart failure, peripheral vascular disease

^b^Risk factors as defined by VISION study [3]: Age ≥ 65 years, history of coronary artery disease, congestive heart failure, PVD, stroke, COPD, active cancer and major general surgery

**Fig. 2:**
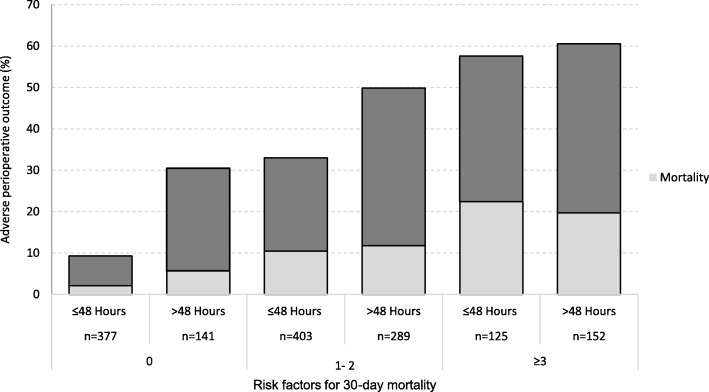
Relationship between risk factors and adverse perioperative outcome by strata of ED-LOS (≤ 48 h versus > 48 h)

Figure 2 shows the association between the number of risk factors and APO by having or not prolonged ED-LOS (> 48 h). Although ED-LOS seems to influence outcomes, the number of risk factors gradually attenuates its effect (from higher among those with no factors, to smaller for those with multiple factors, *p* < 0.001 for interaction). Univariate analysis comparing characteristics of patients who had outcome events with those who did not is shown in Table 3. As expected, patients with APO were older and had more often comorbidities and 30-day mortality risk factors (*p* < 0.01 in all cases). Importantly, patients with APO had longer duration of surgery and 27-h longer ED-LOS (median times 53.2 versus 26.1, *p* < 0.001).

**Table 3:** Patients´ characteristics according to adverse perioperative outcome

	Adverse perioperative outcome		*P*
	Yes (*n* = 519)	No (*n* = 968)	
Gender, male	276 (53.2)	487 (50.3)	0.291
Age (years)	70.2 (59.1–79.4)	61.5 (53.1–72.5)	< 0.001
Medical center			< 0.001
Center A	414 (41.4)	587 (58.6)	
Center B	105 (21.6)	381 (78.3)	
Preoperative medical conditions			
Hypertension	296 (57)	372 (38.5)	< 0.001
Diabetes mellitus	127 (24.5)	131 (13.5)	< 0.001
Chronic renal disease	73 (14.1)	49 (5.1)	< 0.001
Major cardiovascular disease^a^	142 (27.4)	110 (11.4)	< 0.001
COPD	70 (13.5)	49 (5.1)	< 0.001
Active cancer	117 (22.5)	80 (8.3)	< 0.001
Major general surgery	151 (29.1)	85 (8.8)	< 0.001
Risk factors for 30-day mortality^b^			0.006
No risk factors	78 (15)	440 (45.4)	
1 or 2 risk factors	277 (53.4)	415 (45.9)	
≥ 3 risk factors	164 (31.6)	113 (11.7)	
Preoperative interventions			
Medical assessments	13 (6–27)	6 (4–9)	< 0.001
Laboratories	28 (13–59.5)	8 (4–15)	< 0.001
Diagnostic images	4 (2–7)	2 (1–3)	< 0.001
Type of surgery			0.262
General	394 (75.9)	709 (73.2)	
Orthopedic	125 (24.1)	259 (26.8)	
Duration of surgery (hours)	2.2 (1.6–3)	1.6 (1.3–2.3)	< 0.001
ED-LOS (hours)	53.2 (19.5–165.2)	26.1 (13.4–64.9)	< 0.001

Data are expressed as median (interquartile range), or absolute number (%). *COPD* Chronic obstructive pulmonary disease, *ED-LOS* Emergency department length of stay

^a^Includes history of coronary artery disease, stroke, heart failure, peripheral vascular disease

^b^Risk factors as defined by VISION study [3]: Age ≥ 65 years, high risk coronary artery disease, history of PVD, history of stroke, COPD, active cancer and major general surgery

**Fig. 3:**
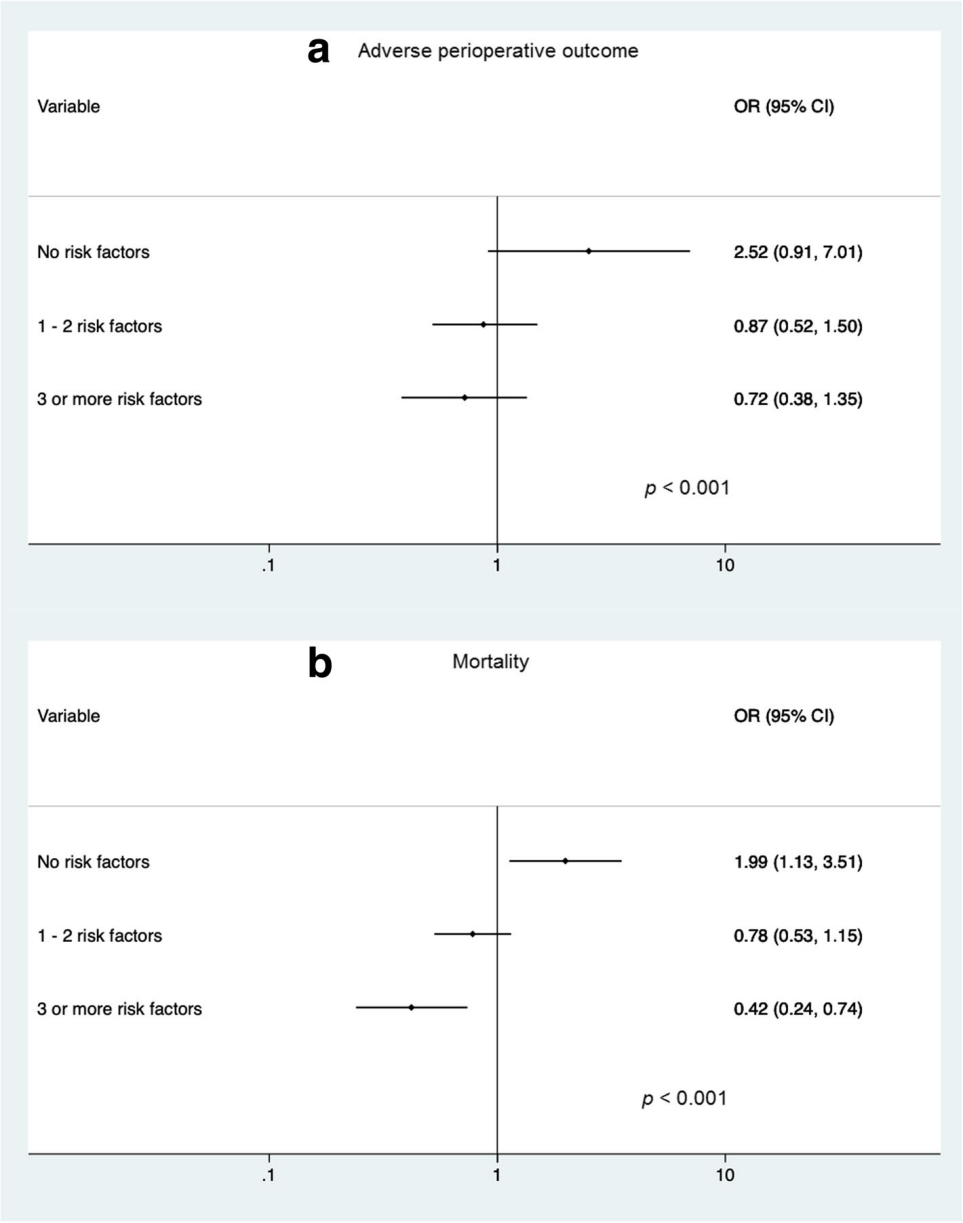
Covariate-adjusted odds ratios for the study outcomes comparing subjects who had a prolonged ED-LOS (> 48 h) compared to those who did not (≤ 48 h) by their levels of risk factors. **a** Adverse perioperative outcome. **b** Mortality

Multivariate analysis indicated that prolonged ED-LOS (> 48 h versus ≤48 h) was independently associated with APO (aOR = 1.99, IC95% 1.13–3.51), as well as other factors: the center performing the surgery; history of renal disease; having 30-day mortality risk factors, the number of ED medical assessments, undergoing abdominal surgery and the duration of surgery (Table 4). Finally, the covariate-adjusted relationship between prolonged ED-LOS and APO significantly decreased by the number of 30-day mortality risk factors present (aORs 2.52, 0.87, 0.72 for having no, 1–2 or 3 30-day mortality risk factors, respectively). Of note, while a high ED-LOS was associated with higher risk of events, this was protective among patients with 3 or more 30-day mortality risk factors (*p* < 0.001 for interaction). A similar trend was observed for mortality (Fig. 3).

**Table 4:** Multivariate analysis of adverse perioperative outcome and mortality

	Unadjusted		Adjusted		*P*
	OR	95% CI	OR	95% CI	
Adverse perioperative outcome					
Prolonged ED-LOS (> 48 h)	2.55	(2.04 - 3.18)	1.99	(1.13 - 3.51)	0.017
Center	2.56	(1.99 - 3.29)	1.55	(1.16 - 2.06)	0.003
Preoperative medical conditions					
Hypertension	2.12	(1.71 - 2.64)	0.97	(0.73 - 1.28)	0.807
Diabetes	2.07	(1.58 - 2.71)	1.19	(0.85 - 1.64)	0.308
Renal Disease	3.07	(2.10 - 4.48)	1.56	(1.01 - 2.43)	0.047
Risk factors for 30-day mortality^a^					
1 or 2 risk factors	3.77	(2.83 - 5.01)	4.20	(2.75 - 6.41)	< 0.001
≥ 3 risk factors	8.19	(5.83 - 11.50)	9.44	(5.50 - 16.2)	< 0.001
Preoperative interventions					
Number of assesments	1.07	(1.06 - 1.08)	1.05	(1.04 - 1.07)	< 0.001
Duration of surgery	1.35	(1.24 - 1.48)	1.20	(1.09 - 1.31)	< 0.001
Mortality					
Prolonged ED-LOS (> 48 h)	1.50	(1.07 - 2.10)	2.52	(0.91 - 7.01)	0.076
Center	2.07	(1.37 - 3.14)	1.28	(0.82 - 2.00)	0.271
Preoperative medical conditions					
Diabetes	1.65	(1.11 - 2.46)	1.19	(0.77 - 1.83)	0.442
Renal Disease	2.87	(1.80 - 4.57)	1.94	(1.16 - 3.24)	0.012
Risk factors for 30-day mortality^a^					
1 or 2 risk factors	3.87	(2.23 - 6.72)	4.63	(2.12 - 10.08)	< 0.001
≥ 3 risk factors	8.31	(4.67 - 14.78)	8.88	(3.83 - 20.59)	< 0.001
Preoperative interventions					
Number of assesments	1.01	(1.01 - 1.02)	1.01	(1.00 -1.01)	0.004
Abdominal surgery	3.72	(2.12 - 6.53)	3.46	(1.93 - 6.20)	< 0.001
Duration of surgery	1.2	(1.09 - 1.33)	1.14	(1.02 - 1.27)	0.019

^a^Risk factors as defined by VISION study [3]: Age ≥ 65 years, history of coronary artery disease, congestive heart failure, PVD, stroke, COPD, active cancer and major general surgery

## Discussion

This study shows that in patients undergoing emergency general or orthopedic surgery, a prolonged (> 48 h) ED-LOS is associated with adverse outcomes. Importantly, the direction of this association varies with patients’ specific aspects such as the number of risk factors for 30-day mortality upon admission to the ED. Patients with no risk factors whose operation began after 48 h of admission were at an elevated risk of perioperative adverse outcome in contrast to patients with three or more risk factors in whom this wait seems to be protective.

The time elapsed to take the patient to surgery can be misleading, subject to interpretation and may be obscured by confounding factors. The field lacks of a standard definition for it, as some authors propose to start measuring from admission to ED, while others from the decision to operate. Furthermore, a variety of medical and non-medical factors can cause delays in transfer patients from the ED to an operating room. The present study suggests that ED-LOS in the more complex cases is justified, as it is used in obtaining an adequate diagnosis and in the preoperative optimization (as shown by receiving more medical assessments and diagnostic workup prior to surgery). Nevertheless, in a low-risk patient, in whom the margin of optimization is limited, prolong ED-LOS would be deleterious and rapid intervention should be prioritized.

The association between delay in surgical intervention and perioperative outcomes has been controversial. In specific areas of emergency general surgery such as perforated peptic ulcer [17] and gastrointestinal perforation [18], times before surgery were reported as a critical determinant of survival. McIsaac et al. showed in a recent propensity matched score analysis that delayed operating room access for ES was also associated with higher in-hospital mortality, longer hospital stays and higher costs. They found that system (i.e., non-medical) factors were the main reasons for delay [19]. However, they not took into account the time from admission to the decision to operate which most of the time is used for diagnostic tests and preoperative therapies in order to optimize patient’s physiologic derangements before surgery. In contrast, several other reports have refuted the association between bad outcomes and surgical delays [20, 21].

In our study, a significant proportion of patients without risk factors (24.2%) remained in the ED for a period of time greater than 48 h before being transferred to operating room. This excess ED-LOS is not explained by preoperative risk factors and therefore raises concern, making this group of patients an excellent target for quality improvement strategies. Furthermore, ED-LOS along with risk stratification should be an indicator of quality of care. Previous research exploring the causes of a prolonged ED LOS in emergent surgery has identified both medical factors (delayed diagnosis) [17] and administrative issues (physicians availability, lack of resources and organizational problems) [8, 19, 20]. We do not know exactly the causes responsible for a prolonged ED-LOS in our system, but we are aware of the need to further evaluate these factors and the specific determinants of negative outcomes. Besides, we would like to assess alternative models of patient care aiming to reduce waiting times and ED LOS [22]. In the meantime, we have implemented several changes focused on the establishment of new standards for access to operating room. Also, a prioritization system has been instituted as well as we have set a surgery room exclusively dedicated to the care of ES. Providing access to the operating room in a timely fashion for patients who need ES will require the efforts and commitment of medical, paramedical and administrative personnel, as well as clear hospital policies that optimize the use of currently available resources.

The results of this study should be read in the context of hospitals located in a middle-income country. Previous research, in general, originates from Canada, the United States and Europe, and the comparison of our results with these studies is relatively difficult due to the differences in the model of patient care between countries. Several investigations have reported international differences in access to surgical care, the ratio of emergency/elective surgeries and the mortality rates [10, 23]. In the present study the median ED-LOS was 24.6 h, with almost 40% of patients waiting for more than 48 h, well in contrast with times found in more advanced countries [17, 18, 24, 25]. This difference is even more relevant given the fact that in developing countries, ES represents a relatively high fraction of the surgical procedures [9].

There are certain limitations in our investigation that need to be highlighted. First, this is a retrospective study with the inherent limitations of this type of design. Second, the results were not discriminated according to the type of surgery; it is clear that for some specific surgical procedures such as appendectomy the surgical intervention should not be delayed for more than 6 h surgery, and also the early intervention in hip fracture is associated with improved outcomes. Finally, our study did not attempt to isolate the reasons for a prolonged ED-LOS because some possibly related variables were not available.

## Conclusions

The results of this study suggest that in emergency abdominal and orthopedic surgery, a prolonged ED-LOS may be associated with an excess of adverse outcomes. This association, however, depends on the number of preoperative risk factors, being harmful for those at low-risk. Further research is needed to identify the causes of prolonged ED-LOS and the specific determinants of adverse perioperative outcomes.

## Abbreviations

APO: Adverse perioperative outcome
ED: Emergency department
ED-LOS: Emergency department length of stay
ES: Emergency surgery
